# Restoration of species‐rich grasslands by transfer of local plant material and its impact on species diversity and genetic variation—Findings of a practical restoration project in southeastern Germany

**DOI:** 10.1002/ece3.8029

**Published:** 2021-08-22

**Authors:** Franziska Kaulfuß, Christoph Reisch

**Affiliations:** ^1^ Institute of Plant Sciences University of Regensburg Regensburg Germany

**Keywords:** genetic variation, hay, local plant material, molecular marker, restoration, species‐rich grassland, threshed plant material, transfer

## Abstract

Restoration of species‐rich grasslands is a key issue of conservation. The transfer of seed‐containing local plant material is a proven technique to restore species‐rich grassland, since it potentially allows to establish genetically variable and locally adapted populations. In our study, we tested how the transfer of local plant material affected the species diversity and composition of restored grasslands and the genetic variation of the typical grassland plant species *Knautia arvensis* and *Plantago lanceolata*.

For our study, we selected fifteen study sites in southeastern Germany. We analyzed species diversity and composition and used molecular markers to investigate genetic variation within and among populations of the study species from grasslands that served as source sites for restoration and grasslands, which were restored by transfer of green hay and threshed local plant material.

The results revealed no significant differences in species diversity and composition between grasslands at source and restoration sites. Levels of genetic variation within populations of the study species *Knautia arvensis* and *Plantago lanceolata* were comparable at source and restoration sites and genetic variation among populations at source and their corresponding restoration sites were only marginal different.

Our study suggests that the transfer of local plant material is a restoration approach highly suited to preserve the composition of species‐rich grasslands and the natural genetic pattern of typical grassland plant species.

## Graphical abstract

In our study, we analyzed the impact of restoration on the genetic variation of typical grassland plant species. Our results indicate that the transfer of local seed‐containing plant material is a technique highly suitable to create genetically variable and locally adapted plant populations.

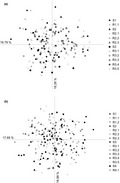

## 1 INTRODUCTION

Species‐rich and extensively managed grasslands declined drastically in central Europe during the recent decades (Poschlod, 2017). Land‐use intensification and abandonment caused an ongoing loss of species diversity since the mid‐twentieth century (Hejcman et al., 2013). On the one hand, higher productivity and mowing frequencies due to increased fertilizer application decreased species richness of grasslands (Jacquemyn et al., 2003; Socher et al., 2012; Zechmeister et al., 2003). Moreover, atmospheric nitrogen deposition caused a general loss of species richness and shifts in species composition of European grasslands (Diekmann et al., 2014; Wesche et al., 2012). On the other hand, the dominance of grasses (Zulka et al., 2014) and litter accumulation (Jensen & Gutekunst, 2003; Piqueray et al., 2015; Ruprecht & Szabó, 2012) due to land‐use abandonment reduced species diversity in grasslands. Consequently, nearly three‐quarters of all grassland plant communities are highly endangered today (Rennwald, 2000). The restoration of species‐rich grasslands is, therefore, a key issue of conservation.

Principally, species‐rich grasslands may be restored by improving habitat conditions, for example, via the reestablishment of traditional management regimes, rewetting, or the removal of nutrients from the soil (Bakker, 1989; Pfadenhauer & Grootjans, 1999). Increasing species richness by these restoration approaches is, however, often limited due to the lack of viable seeds in the soil or the surrounding habitats (Bakker et al., 1996; Bossuyt & Honnay, 2008). After decades of intensive grassland management, soil seed banks are usually depleted (Bakker et al., 1996; Bissels et al., 2005) and the immigration of plants from surrounding grasslands is often complicated by landscape fragmentation and the lack of dispersal vectors (Hölzel et al., 2012). Creating species‐rich grasslands requires, therefore, the introduction of seed material from other sources than the restoration site.

The problem of seed limitation in grassland restoration can be solved in different ways. One possibility is using commercially produced seed mixtures for restoration, which has become a common and comparatively simple approach in recent years (Jongepierová et al., 2007; Török et al., 2010; Walker et al., 2015), since seed mixtures are easily available from a number of different seed producers. Another possibility is the restoration of species‐rich grassland by the introduction of local plant material from source sites via transfer of seed‐containing chaff, threshed plant material, or green hay (non‐dried fresh plant material) (Kiehl et al., 2010). These approaches are more traditional methods that have been applied for centuries and represent proven techniques to create new grasslands (Albert et al., 2019; Coiffait‐Gombault et al., 2011; Kiehl & Wagner, 2006).

The transfer of local plant material allows one to move the species richness of a whole plant community from a source site to a potential restoration site and at the same time to establish genetically variable populations that are locally adapted to specific regions (van der Mijnsbrugge et al., 2010).

The use of local seed material is generally recommended in restoration (van der Mijnsbrugge et al., 2010), since plant populations are adapted to local environmental conditions (McKay et al., 2005). Mixing genetically differing genotypes from geographically different regions may cause a loss of locally adapted genotypes and result in outbreeding depression (Hufford & Mazer, 2003). Co‐adapted gene complexes can be destroyed and local adaptations get lost, which decreases fitness and performance of plant populations (Frankham et al., 2002; Keller et al., 2000; Montalvo & Ellstrand, 2001). Seed material used for restoration should match the gene pool of the populations occurring in the vicinity of the restoration site (McKay et al., 2005), and the transfer of locally harvested plant material is, therefore, considered as the “gold standard” to preserve patterns of genetic variation (Dittberner et al., 2019).

Worldwide, seed production, and seed transfer zones have been defined for the commercial production of local seed mixtures used in ecological restoration to avoid the negative effects of mixing local and nonlocal genotypes (Krauss et al., 2013; Miller et al., 2011). In recent years, genetic differentiation among populations from different seed transfer zones (Bucharova et al., 2017; Listl et al., 2017) and the impact of sowing local seeds on the genetic variation of grassland species have been studied intensively (Aavik et al., 2012; Kaulfuß & Reisch, 2019; Reiker et al., 2015). The impact of transferring local plant material on patterns of genetic variation has, however, been hardly analyzed (Dittberner et al., 2019; Van Rossum et al., 2020).

Generally, the restoration process has a strong impact on genetic variation (Mijangos et al., 2015). Previous studies comparing source populations and restored populations of different species often revealed decreased levels of genetic variation in restored populations (Aavik et al., 2012; Vandepitte et al., 2012), although this was not always the case (Dittberner et al., 2019; Kaulfuß & Reisch, 2019). The observed loss of genetic variation within populations may be caused by bottlenecks occurring during seed harvesting and seed production or by founder effects during recolonization or due to the origin of seeds (Mijangos et al., 2015). Such bottlenecks or founder effects may also occur during grassland restoration by the transfer of seed‐containing local plant material. In particular, the collection of the material at the source site and the establishment of plants from the seeds at the restoration site are critical steps (Kiehl et al., 2010), potentially limiting the size of the restored populations and consequently also the genetic variation within these populations. Furthermore, the potentially reduced number of transferred individuals and the geographic distance between the selected locations may cause genetic drift increasing variation among populations from source and restoration sites (Kaulfuß & Reisch, 2019).

In this study, we investigated the impact of grassland restoration by the transfer of green hay and threshed plant material in southeastern Germany on species diversity and composition of the restored grasslands and the genetic variation of the typical central European grassland species *Knautia arvensis* and *Plantago lanceolata*. More specifically, we asked the following questions: (a) Are grasslands at the source and restored sites comparable in species composition and diversity? (b) Is the level of genetic variation within populations of the study species differing between source populations and restored populations? (c) How high is genetic variation among source populations and restored populations of the study species? (d) Is the transfer of green hay and threshed plant material an effective tool in conservation to restore species‐rich and genetically diverse grasslands?

## 2 MATERIAL AND METHODS

### 2.1 Study area and sites

**FIGURE 1:**
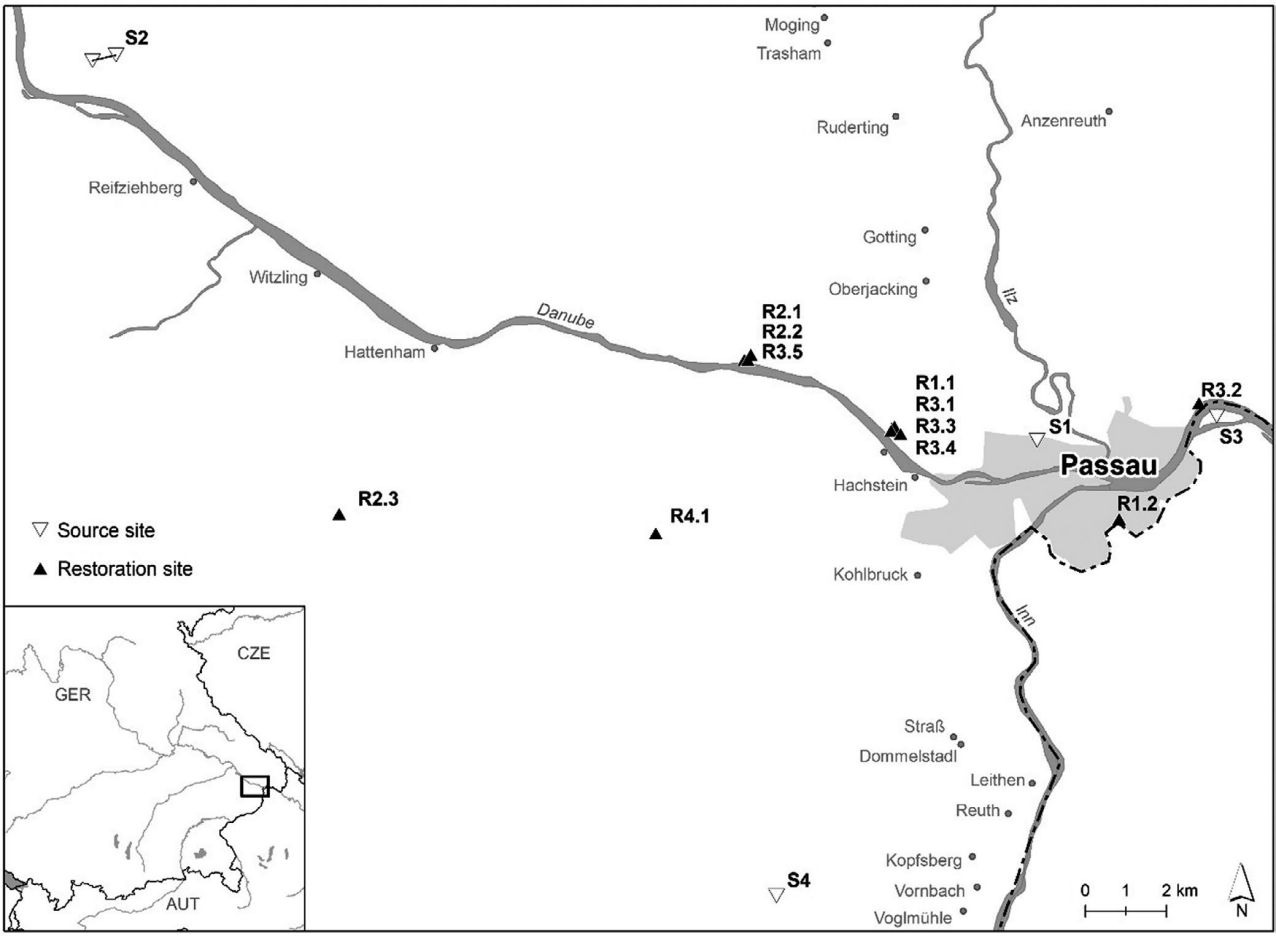
Geographic position of the study sites near Passau in southeastern Germany. Source sites are marked with upside‐down, white triangles. Restoration sites are indicated by black triangles. Restoration sites (R1.1–R4.1) are labeled so that the first number corresponds to the source location (S1–4) and the second number corresponding to the replicate (R1.2 corresponds to the 2nd replicate of restoration site sourced from S1)

For our study, we selected grasslands at 15 study sites in southeastern Germany near Passau (Figure 1; Table 1). At four of these sites (S1–S4), local plant material was gathered from species‐rich grasslands between 2005 and 2014. The plant material from these source sites was then used to establish grasslands on former arable fields at eleven restoration sites (R1.1–R4.1). The variation in the numbers of restoration sites to source sites is due to the fact that our study was part of a practical restoration project by the landscape conservation association of Passau. Seed‐containing plant material was obtained by mowing the grasslands at the source sites in June and by threshing the grasslands with an automatic harvester in August. Green hay and threshed plant material were then transferred from S1 to R1.1–R1.2, from S2 to R2.1–R2.3, from S3 to R3.1–R3.5, and from S4 to R4.1. At the restoration sites, topsoil was removed to reduce soil fertility and the number of seeds from the previous vegetation in the soil seed bank (Rasran et al., 2007) before the local plant material was spread.

**TABLE 1:** Study sites with number (No.), site type (S: source or R: restoration site), geographic position (Lat. and Lon.), area of study site (m²), species diversity (*SD*), and the year of restoration

No.	Type	Lat.	Lon.	Area [m²]	*SD*	Year
S1	S	48.583152	13.452477	8,000	61	–
S2	S	48.672036	13.146660	12,400	86	–
S3	S	48.587754	13.512742	34,600	82	–
S4	S	48.483265	13.362789	19,500	55	–
Mean source sites					71	
R1.1	R	48.586844	13.404991	16,000	59	2011
R1.2	R	48.565453	13.479381	11,000	52	2008
R2.1	R	48.602194	13.356277	8,000	75	2009
R2.2	R	48.603368	13.357393	8,000	73	2009
R2.3	R	48.569620	13.218924	10,200	71	2012
R3.1	R	48.586531	13.404060	6,000	51	2008
R3.2	R	48.590790	13.506841	2,400	77	2005
R3.3	R	48.586004	13.403802	3,300	56	2012
R3.4	R	48.585256	13.406871	8,100	58	2010
R3.5	R	48.602306	13.355209	4,000	51	2008
R4.1	R	48.564128	13.324489	2,500	38	2014
Mean restoration sites					60	

### 2.2 Species diversity and composition of grasslands

At each study site, species diversity and composition of the selected grasslands were assessed. Therefore, all species occurring in the grasslands were identified and registered while walking across the study sites in the vegetation period 2016 (Heinz et al., 2012).

### 2.3 Study species and sampling of plant material

For genetic analyses, we selected the two widespread and outcrossing grassland species *Knautia arvensis* (Coult) and *Plantago lanceolata* (L.). *K. arvensis* is a hemicryptophytic, perennial plant species, belonging to the Caprifoliaceae family (Oberdorfer, 2001). Its distribution ranges from North‐West Africa to Asia and Europe. The plant occurs in fertile meadows, semiarid grasslands, waysides, forest edges, and extensively used fields (Rothmaler, 2005). The species forms a basal rosette and paired stem leaves are spear‐shaped. Growth height is between 25 and 100 cm. The species may be diploid (2n = 20) or tetraploid (2n = 40), as previously reported (Kaulfuß & Reisch, 2019). In the study presented here, all individuals were tetraploid, as detected by flow cytometry (Dolezel et al., 2007). *K. arvensis* flowers between July and September and is insect‐pollinated by bees, bumblebees, butterflies, syrphid‐flies, and wasps (Oberdorfer, 2001). *P. lanceolata* is a hemicryptophytic, perennial plant species, belonging to the Plantaginaceae family (Oberdorfer, 2001). The species is widespread all over Europe from the Iberian peninsula to central Asia and occurs in fertile meadows and fields and on loamy or sandy soils (Rothmaler, 2005). The species is a perennial rosette‐forming herb with lanceolate spreading leaves. Flower stems are 10–40 cm high, leafless, hairy and have ovoid inflorescences with many small flowers (Oberdorfer, 2001). *Plantago lanceolata* is diploid (2n = 12) (Oberdorfer, 2001). Consequently, in our study, all individuals exhibited the same diploid ploidy level, as revealed by flow cytometry (Dolezel et al., 2007). *P. lanceolata* is mainly wind‐pollinated, but insect pollination by short‐proboscic bees, flies, beetles, and syrphid‐flies is also possible (Oberdorfer, 2001).

In early summer of 2016, young rosette leaves of the study species were sampled in situ from 16 individuals per population in grasslands at source sites and restored sites and dried in teabags over silica gel for further investigation. The number of sampled populations differed slightly between the study species since *K. arvensis* did not occur at all study sites. In total, we sampled plant material of *K. arvensis* from populations at three source sites and nine corresponding populations at the restoration sites. *P. lanceolata* occurred at all study sites, and we collected, therefore, plant material from populations at four source sites and eleven corresponding populations at the restoration sites (Table 1). At all study sites, the population size of *K. arvensis* and *P. lanceolata* was determined by counting the number of individuals in 10 randomly placed one‐square‐meter grids (Reisch et al., 2018). The mean number of individuals per square meter was then multiplied with the area of the grassland to calculate population size (Table 1).

### 2.4 Molecular analysis

For DNA isolation, the cetyltrimethylammonium bromide (CTAB) protocol by Rogers and Bendich (1994) with adaptions by Reisch (2007) was applied. For every sample, first the concentration of genomic DNA was quantified with a microvolume spectrometer (NanoDrop One, Thermo Scientific), and afterward, a dilution with a standardized concentration of 7.8 ng/µl was prepared. Genome‐wide genotyping with amplified fragment length polymorphisms (Vos et al., 1995) was used to assess genetic variation. AFLPs were performed, following the Beckman Coulter protocol as described before (Bylebyl et al., 2008). Primers for selective PCR were chosen, according to Kaulfuß and Reisch (2019). The primer combinations for *K. arvensis* were *Mse*I‐CAG/*Eco*RI‐ACC (D2), *Mse*I‐CTT/*Eco*RI‐AGG (D3), and *Mse*I‐CTT/*Eco*RI‐ACT (D4). Primer combinations for *P. lanceolata* were *Mse*I‐CTG/*Eco*RI‐AGC (D2), *Mse*I‐CAA/*Eco*RI‐AGG (D3), and *Mse*I‐CAG/*Eco*RI‐ACA (D4). *Eco*RI primers were labeled with fluorescent dyes for fragment detection (Beckman dye D2, D3, and D4). DNA fragments were separated by size with capillary gel electrophoresis performed on an automated sequencer (GeXP, Beckmann Coulter). The results were exported as .crv files. AFLP fragment patterns were evaluated using the software Bionumerics 4.6 (Applied Maths, Kortrijk, Belgium). Each strong and clearly defined DNA fragment was classified as present (1) or absent (0) to create a binary (0/1) matrix, which was the basis for further statistical analysis. We repeated about 10% of the samples and calculated a genotyping error rate (Bonin et al., 2004), which was 4.3% for *K. arvensis* and 5.6% for *P. lanceolata*.

### 2.5 Statistical analysis

Based upon the species occurrence list, species diversity was calculated for each site as number of occurring species. We used a one‐way ANOVA to test whether species diversity differed significantly between source and restoration sites and Spearman's rank correlation coefficients to check whether species diversity depended on the year of restoration. All tests were done in IBM Statistics 24 for Windows, IBM Corporation.

Furthermore, we estimated the degree of floristic (dis)similarity in vegetation composition between the source and restoration sites. We performed a nonmetric multidimensional scaling (NMDS) with presence–absence data based on Sorensen similarity index using PC‐ORD version 7 software (McCune & Mefford, 2016). The NMDS ordination was performed with 50 runs of real data and 50 randomized (by row) runs with a stability criterion of 0.00001 and a maximum of 200 iterations. Standard stepdown procedures were used to find the appropriate number of axes sufficient to reduce stress, which measures how well the distance ordination space corresponds to the dissimilarity in species composition. A multiresponse permutation procedure (MRPP) implemented in PC‐ORD version 7 software (McCune & Mefford, 2016) was used to test for differences between the two groups.

Genetic variation within the populations of *K. arvensis* and *P. lanceolata* was calculated as Nei's gene diversity (*H*) with the program AFLPsurv (Vekemans, 2002). Population size and genetic variation within populations from source and restoration sites were compared using one‐way ANOVAs. Spearman's rank correlation coefficients were computed to test for correlation between Nei's gene diversity and age of the grasslands as well as population size of *K. arvensis* and *P. lanceolata* at the study sites. All tests were done in IBM Statistics 24 for Windows, IBM Corporation.

The program Structure version 2.3.4 (Pritchard et al., 2000, 2007) was used to perform Bayesian cluster analysis. This method enables to examine population structure in the data set and assign individuals into groups without prior definition of populations. The presumable number of groups was computed using 10,000 Markov chain Monte Carlo (MCMC) simulations and a burn‐in period of 100,000 iterations. Analyses for the predefined value of *K* were run 20 times per *K* = 1–16 for *K. arvensis* and 20 times per *K* = 1–18 for *P. lanceolata* (Falush et al., 2003, 2007). Results were summarized with the program Structure Harvester (Earl & Vonholdt, 2012). Group assignment was an ad hoc quantity procedure calculating Δ*K* (Evanno et al., 2005).

The software GenAlEx 6 (Peakall & Smouse, 2006) was employed to analyze patterns of genetic similarities between individuals. Therefore, a principal coordinate analysis (PCoA) based on a squared Euclidean distance matrix was calculated. Furthermore, the program was used to compute analyses of molecular variance, AMOVAs (Excoffier et al., 1992), to investigate genetic differentiation between populations on source and restored sites.

## 3 RESULTS

### 3.1 Species diversity and composition

**FIGURE 2:**
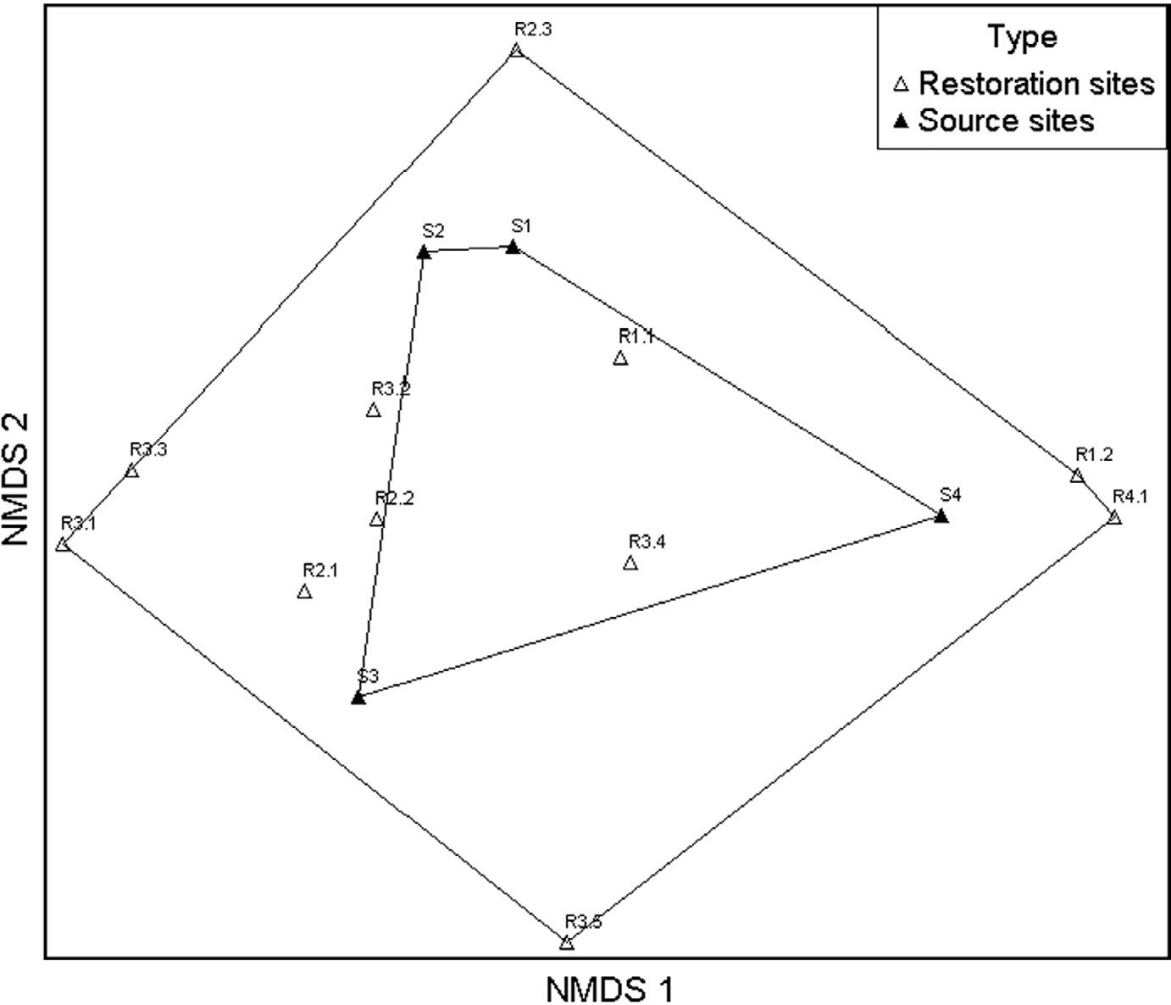
Nonmetric multidimensional scaling (NMDS) of the study sites based on Sorensen similarity index. Grasslands at source sites (upside‐down, white triangles) were more similar in their species composition to each other than grasslands at restoration sites (black triangles). S = source site, R = restored site; numbers indicate transfers belonging together (i.e., S1 and R1.1)

In total, we observed 165 plant species in the grasslands at all study sites (Table A1). They contained many protected (*Centaurium umbellatum*, *Dianthus armeria*, *Dianthus carthusianorum*, *Dianthus deltoides*, *Primula elatior*) and red list species (*Agrostema githago*, RL 1; *Astragalus cicer*, RL 3; *Linum perenne*, RL 1). On average, we identified 63 plant species per grassland. At source sites, the number of plant species varied between 55 and 86 with a mean of 71 species (Table 1), while the number of plant species at restoration sites ranged from 38 to 77 with a mean of 60 species (Table 1). However, species diversity did not differ significantly between source and restoration sites (one‐way ANOVA, *p* = .178) and did not depend on the year of restoration (Spearman correlation, *r* = −0.194, *p* = .568). NMDS revealed differences in species composition between grasslands at source and restoration sites (Figure 2), but MRPP indicated that these differences were not statistically significant (*A* = −0.008, *T* = 0.47, *p* = .63). Grasslands were more similar to each other at source sites than at restoration sites. However, the plots originating from the same source site were not grouped together.

### 3.2 Genetic variation within populations

For *K. arvensis,* AFLP analysis resulted in 127 fragments. 78.57% of the fragments were polymorphic. In populations at source sites, Nei's gene diversity (*H*_Ka_) varied between 0.27 and 0.30 with a mean of 0.29. In populations at restoration sites, *H*_Ka_ ranged from 0.22 to 0.30 with a mean of 0.28 (Table 2). Nei's gene diversity was not significantly different between populations at source and restoration sites (one‐way ANOVA, *p* = .835). Population size (PS) of *K. arvensis* (PS_Ka_) differed significantly between source and restoration sites (one‐way ANOVA, *p* = .000), but we observed no significant correlation between *H*_Ka_ and population size (PS) or year (Y) of restoration (Spearman correlation, *r_Ka_PS_* = −.222*, p_Ka_PS_* = .489; *r_Ka_Y_* = .202*, p_Ka_Y_* = .603).

**TABLE 2:** Genetic variation within populations of *K. arvensis* and *P. lanceolata* measured as Nei's gene diversity (*H*_Ka_ and *H*_Pl_) at source sites and the corresponding restoration sites, with number (No.), site type (S: source or R: restoration site), population size (PS_Ka_ and PS_Pl_), and number of analyzed individuals (N_Ka_ and N_Pl_)

No.	Type	PS_KA_	PS_Pl_	N_Ka_	*H*_Ka_	N_Pl_	*H*_Pl_
S1	S	16,800	51,200	16	0.29	16	0.31
S2	S	28,520	16,120	16	0.30	16	0.32
S3	S	31,140	83,040	15	0.27	15	0.32
S4	S	–	79,950	–	–	16	0.32
Mean source sites		25,487	57,578	15.6	0.29	15.7	0.32
R1.1	R	3,200	81,600	15	0.22	15	0.35
R1.2	R	–	92,400	–	–	15	0.31
R2.1	R	7,200	20,000	15	0.30	16	0.30
R2.2	R	8,000	29,600	16	0.28	16	0.29
R2.3	R	3,060	45,900	15	0.30	16	0.28
R3.1	R	3,600	18,600	15	0.28	13	0.34
R3.2	R	1,680	16,320	16	0.29	15	0.33
R3.3	R	3,630	18,810	15	0.29	14	0.35
R3.4	R	1,620	59,130	14	0.30	13	0.37
R3.5	R	2,400	26,400	14	0.29	16	0.29
R4.1	R	–	12,250	–	–	16	0.30
Mean restoration sites		3,821	38,274	15.0	0.28	15.0	0.32
*p* (one‐way ANOVA)		.**000**	.346		.835		.830

For *P. lanceolata*, 122 fragments could be detected and 90.35% of the fragments were polymorphic. In populations at source sites, Nei's gene diversity (*H*_Pl_) ranged from 0.31 to 0.32 with a mean of 0.32 (Table 2). In populations at restoration sites, *H*_Pl_ varied between 0.28 and 0.37 with a mean of 0.32. We detected no significant differences between populations at source and restoration sites (one‐way ANOVA, *p* = .830). Population size (PS) of *P. lanceolata* (PS_Pl_) differed not significantly between source and restoration sites (one‐way ANOVA, *p* = .346), and we observed also no significant correlation between Nei's gene diversity and population size (PS) or year (Y) of restoration (Spearman correlation, *r_Pl_PS_* = −.033*, p_Pl_PS_* = .910; *r_Pl_Y_* = .012, *p_Pl_Y_* = .973).

### 3.3 Genetic variation among populations

**FIGURE 3:**
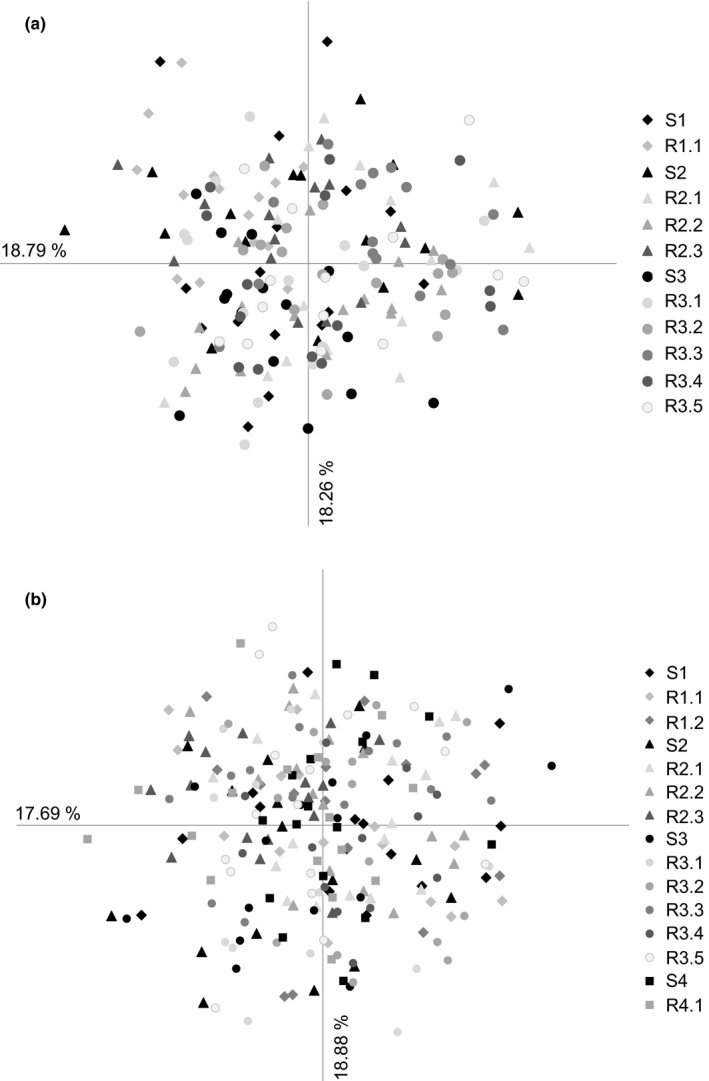
Principal coordinate analyses (PCoA) for (a) *K. arvensis* and (b) *P. lanceolata*. For both species, all investigated populations were admixed and no grouping of individuals according to population, site type, or geographic position could be detected. Population labels follow Table 1

**FIGURE A1:**
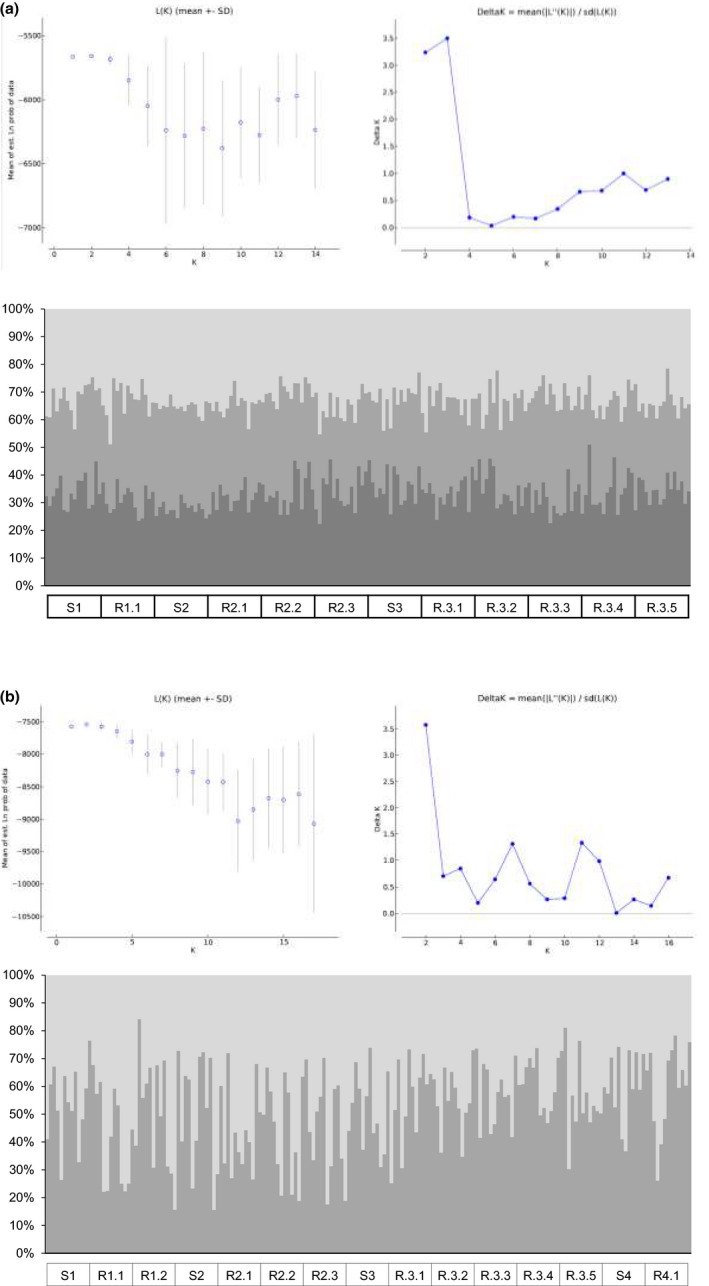
Results of Bayesian cluster analysis for (a) *Knautia arvensis and* (b) *Plantago lanceolata*. Different shades of grea classify the received groups. Given are Ln P(D) variance, Delta *K*, and bar plot diagram. *K* for the tested groups is (a) *K* = 1–16 (*K. arvensis*) and (b) *K* = 1–20 (*P. lanceolata*)

For *K. arvensis*, the principal coordinate analysis (Figure 3a) revealed one group comprising all individuals without any separation of individuals according to population, site type, or geographic position of the investigated populations. For the Bayesian cluster analysis, Evanno's delta *K* approach indicated that the populations formed 3 genetic clusters (Figure A1a). However, the assignment plots produced for *K* = 3 showed no distinct grouping by population, site type, or geographic position. Based on the high *L*(*K*) values at *K* = 1–3, it is likely that *K* = 3 is over clustering these data, and only one genetic cluster is present. In the AMOVAs (Table A2), molecular variance among all populations was generally low (Φ_PT_ = 0.04). Source and restoration sites differed only weakly from each other (Φ_PT_ = 0.04). Molecular variance among populations at source sites was, however, slightly higher (Φ_PT_ = 0.06) than among populations at restoration sites (Φ_PT_ = 0.03). Comparing genetic variance between source and restoration sites for each transfer separately revealed Φ_PT_ values between 0.004 and 0.07 (Table A2). Six of nine transfers resulted in nonsignificant differentiation or a molecular variance below a Φ_PT_ of 0.04.

Principal coordinate analysis (Figure 3b) also revealed one group without any separation of individuals according to population, site type, or geographic position of the investigated populations for *P. lanceolata*. For the Bayesian cluster analysis, Evanno's delta *K* approach indicated that the populations formed 2 genetic clusters (Figure A1b). However, given that there are no observable clustering patterns with the assignment plot, the fact that Evanno's delta *K* is biased toward *K* = 2 and that the *L*(*K*) also has a high value at *K* = 1, it is likely that all individuals also cluster into one group.

The AMOVAs (Table A3) revealed a very low level of molecular variance among all populations (Φ_PT_ = 0.02) as well as between source and restoration sites, among populations from source sites or among populations from restoration sites (all Φ_PT_ = 0.02). Comparing genetic variance between source and restoration sites for each transfer separately resulted again in very low levels of molecular variance varying from 0.002 to 0.05 (Table A3). Eight of eleven transfers resulted in nonsignificant differentiation or a molecular variance below a Φ_PT_ of 0.03.

## 4 DISCUSSION

### 4.1 Impact of restoration on species diversity and composition

In our study, we observed no significant differences in species diversity and composition between grasslands at source and restoration sites, which supports the conclusion of previous studies that the transfer of plant species via seed‐containing chaff, threshed plant material, or green hay (Kiehl et al., 2010) is generally a promising approach to restore species‐rich grasslands (Albert et al., 2019; Coiffait‐Gombault et al., 2011; Kiehl & Wagner, 2006). The establishment of a large proportion of species occurring at a source site in restored grasslands requires, however, much effort and the repeated transfer of plant material (Kiehl & Wagner, 2006). Species diversity may, hence, for practical reasons be lower at restored than at source sites (Kiehl & Pfadenhauer, 2007), a trend we also observed since mean species diversity was slightly lower in grasslands at restored sites than at source sites, although the difference was not significant.

Successful grassland restoration with local plant material depends, in particular, on harvesting time and soil preparation (Bischoff et al., 2018; Schmiede et al., 2012). Depending on species phenology, the composition of seeds within the local plant material used for restoration is strongly affected by harvesting time. Multiple transfers of plant material collected at different times are, therefore, suggested to achieve high transfer rates and similar species composition (Kiehl & Wagner, 2006). The grasslands we studied here were restored over a longer period of time and the harvesting process varied for practical reasons seasonally and between years, which may have contributed to the observed differences in species diversity and composition among grasslands from source and respective restoration sites.

### 4.2 Impact of life‐history traits and restoration on genetic variation

In our study, we observed clear differences in genetic variation between the study species. *K. arvensis* and *P. lanceolata* exactly reflected the pattern of genetic variation, which has previously been reported for other wind‐pollinated and more frequently distributed plant species compared to insect‐pollinated and less frequently distributed species. Generally, the former exhibit higher levels of variation within populations but lower variation among populations, whereas the latter show lower levels of variation within but stronger variation among populations (Reisch & Bernhardt‐Römermann, 2014). Moreover, populations of *P. lanceolata* were much larger than populations of *K. arvensis*, which may also contribute to the higher level of genetic variation within populations of *P. lanceolata* compared to *K. arvensis* (Leimu et al., 2006). Hence, the results of our study corroborate the strong impact of life‐history traits and population size on the genetic variation of plant species, which has been reported in previous studies.

Besides life‐history traits and population size, genetic variation within and among plant populations may, however, also be influenced by restoration, in particular by bottlenecks caused during the harvesting process (Mijangos et al., 2015). First, the number of seeds harvested for restoration may be limited for practical reasons. It is, for instance, generally recommended not to harvest the whole source site but only two‐thirds of the area. Secondly, the number of transferred seeds and their origin may be affected by plant phenology, since not all individuals fruit at the same time. This means that seed harvesting often comprises not all individuals at the source site but only a subset. Consequently, only a part of the available gene pool is transferred, which may cause a bottleneck and hence reduced levels of genetic variation within restored populations.

Genetic analyses revealed similar levels of genetic variation within populations of *P. lanceolata* from source and restoration sites, which was to be expected due to similar population size. Interestingly we also observed no significant differences in genetic variation between populations of *K. arvensis* despite significantly smaller population size at restoration sites than at source sites. This may be explained in two ways. First, the lack of differences may be a statistical bias due to the low number of *K. arvensis* populations at source sites in our study, which may obscure potential differences in genetic variation. Genetic variation was slightly but not significantly lower in populations from restoration sites, which may support this assumption. Second, high immigration rates, or more specifically gene flow, from nearby grassland sites may have caused a fast recovery of genetic variation within recently founded populations (Tremetsberger et al., 2003). Previous studies of different species often revealed decreased levels of genetic variation in restored compared to source populations (Aavik et al., 2012; Vandepitte et al., 2012), although this must not be necessarily the case (Kaulfuß & Reisch, 2019). Genetic variation within populations restored by transfer of local plant material has hardly been analyzed, but Dittberner et al. (2019) reported, similar to our results, also no loss of genetic variation in floodplain meadow populations of *Arabis sagittata* and *A. nemorensis* restored by hay transfer. This supports our perception that the transfer of local, seed‐containing plant material is a restoration approach, which is suitable to restore genetically variable populations.

Restoration by transfer of local plant material may not only reduce genetic variation within populations but also increase genetic differentiation among populations. Seed harvesting may comprise for seasonal or practical reasons not all individuals at the source site, which means that not the full gene pool available is transferred but only a subset. Moreover, seedling establishment at the restoration site may represent a filter selecting specific genotypes. Both could result in increased levels of genetic differentiation among populations from source and restoration sites. In our study, genetic differentiation among populations of both *K. arvensis* and *P. lanceolata* from source and restoration sites was much lower than reported for other common, outcrossing plant species (Reisch & Bernhardt‐Römermann, 2014). Even more important is, however, that genetic differentiation among source sites and restoration sites was lower (*K. arvensis*) or equivalent (*P. lanceolata*) to genetic differentiation among populations from source sites. These results support the assumption that—at least for the investigated populations in our study—grassland restoration by transfer of green hay and threshed plant material caused neither a decrease in genetic variation within nor an increasing divergence among populations at source and restored sites. This again supports our assessment that the transfer of local plant material seems to be an approach, which allows to restore genetically comparable grassland populations. Our study underpins, therefore, the perception that the transfer of local plant material is indeed the restoration approach most suitable to preserve the natural genetic pattern of plant species.

## CONFLICT OF INTEREST

The authors declare that they have no conflict of interest.

## AUTHOR CONTRIBUTIONS

**Franziska Kaulfuß:** Investigation (lead); writing‐original draft (equal). **Christoph Reisch:** Conceptualization (lead); writing‐original draft (equal).

## Data Availability

Data are available as Appendix A. AFLP fingerprints are provided by the authors upon request.

## APPENDIX A

**TABLE A1:** List of species occurring in the grasslands at the selected study sites

Study site	S1	R1.1	R1.2	S2	R2.1	R2.2	R2.3	S3	R3.1	R3.2	R3.3	R3.4	R3.5	S4	R4.1
*Achillea millefolium*	1	1	0	1	1	1	1	1	1	1	1	1	1	1	1
*Aegopodium podagraria*	0	0	0	0	0	0	0	1	0	0	0	0	0	0	0
*Agrimonia eupatoria*	0	1	0	1	0	0	1	0	0	0	0	0	0	1	0
*Agrostema githago*	0	0	0	0	0	0	0	0	1	0	0	0	0	0	0
*Agrostis capillaris*	1	1	1	1	1	1	1	1	0	0	0	1	1	1	1
*Agrostis giganthea*	0	0	0	0	0	0	0	0	0	0	0	0	0	1	0
*Agrostis stolonifera*	0	0	0	0	0	0	0	1	0	0	0	0	0	0	0
*Ajuga reptans*	0	0	1	0	0	0	0	0	0	0	0	0	0	1	0
*Alchemilla vulgaris*	0	0	1	0	0	0	0	0	0	0	0	0	0	0	0
*Allium carinatum*	0	0	0	1	0	0	0	0	0	0	0	0	0	0	0
*Allium oleraceum*	1	0	0	0	0	0	0	0	0	0	0	0	0	0	0
*Alopecurus pratense*	1	0	0	0	0	0	0	0	0	0	0	0	0	1	1
*Anthoxanthum odoratum*	1	1	1	1	1	1	1	1	0	1	1	1	1	1	1
*Anthriscus cerefolium*	0	0	0	0	0	0	0	1	0	0	0	0	0	0	0
*Aquilegia spec*.	0	0	0	0	1	0	0	1	0	0	0	0	0	0	0
*Arabis hirsuta*	0	0	0	1	0	1	0	0	0	0	0	0	0	0	0
*Arenaria serpyllifolia*	0	0	0	0	0	0	0	1	0	0	0	0	0	0	0
*Arrhenatherum elatius*	1	1	1	1	1	1	1	1	1	1	1	1	1	1	1
*Artemisia vulgaris*	0	1	0	0	0	0	0	0	0	0	0	0	0	0	0
*Astragalus cicer*	0	0	0	0	1	0	0	0	0	0	0	0	0	0	0
*Astragalus spec*.	0	0	0	0	0	0	0	1	0	0	0	0	0	0	0
*Bellis perennis*	0	0	0	0	0	0	0	1	0	0	0	0	0	0	0
*Betonica officinalis*	1	1	1	1	0	0	1	0	0	1	1	1	0	0	0
*Brachypodium sylvaticum*	0	0	0	0	0	0	0	0	0	1	0	0	0	0	0
*Briza media*	1	0	1	1	1	1	1	1	1	1	1	1	1	1	1
*Bromus erectus*	0	0	0	1	1	0	1	1	1	1	1	1	0	0	0
*Calamagrostis epigejos*	1	0	0	0	0	0	0	1	0	1	0	0	0	1	0
*Calliergonella cuspidata*	0	1	0	0	1	1	0	0	1	0	1	1	1	1	1
*Campanula glomerata*	0	0	0	0	1	1	0	1	1	1	1	0	1	0	0
*Campanula patula*	1	1	1	1	1	1	1	0	0	1	0	1	0	1	1
*Campanula rapunculus*	0	0	0	0	0	0	0	0	0	0	1	1	0	0	0
*Campanula rotundifolia*	0	0	0	0	0	1	1	0	0	0	0	0	0	0	0
*Carec flacca*	0	0	0	1	0	0	0	0	0	0	0	0	0	0	0
*Carex hirta*	0	0	1	0	0	1	0	1	0	0	0	0	1	0	0
*Carex pallescens*	0	0	0	1	0	0	0	0	0	0	0	0	0	0	1
*Centaurea jacea*	1	1	1	1	1	1	1	1	1	1	1	1	1	1	1
*Centaurea nigra*	0	0	0	1	1	0	0	0	0	0	0	0	0	0	0
*Centaurea scabiosa*	0	0	0	0	0	0	1	0	0	0	0	0	0	0	0
*Centaurium umbellatum*	0	1	0	1	1	1	0	1	1	1	1	1	1	0	0
*Cerastium holosteoides*	0	0	1	1	1	1	0	1	1	1	1	1	1	0	1
*Cirsium oleraceum*	0	0	0	0	0	0	0	1	0	0	0	0	1	0	0
*Cirsium vulgare*	0	1	0	0	1	1	0	1	0	0	0	0	0	0	0
*Cisium arvensis*	0	0	0	1	0	0	0	1	0	0	0	0	1	0	0
*Clinopodium vulgare*	0	1	0	0	1	0	0	0	0	0	0	1	0	0	0
*Colchicum autumnale*	0	0	0	1	0	0	0	1	1	0	0	0	1	1	0
*Convolvulus arvensis*	1	1	1	1	1	0	1	0	1	1	0	0	1	1	0
*Coronilla varia*	0	1	0	1	1	1	1	1	1	1	1	0	1	0	0
*Crepis biennis*	1	1	1	0	1	1	1	1	1	1	1	1	1	1	1
*Cynosurus cristatus*	0	1	1	1	0	0	1	1	0	1	0	0	0	1	1
*Dactylis glomerata*	1	1	1	1	1	1	1	1	1	1	1	1	1	1	1
*Daucus carota*	1	1	0	1	1	1	1	1	1	1	1	0	1	1	0
*Dianthus carthusianorum*	1	0	0	1	1	1	1	0	1	0	1	0	0	0	0
*Dianthus deltoides*	0	0	1	1	0	1	1	0	0	1	0	1	0	1	0
*Diantus armeria*	0	0	0	1	0	1	0	0	0	0	0	0	0	0	0
*Echium vulgare*	0	0	0	0	0	0	0	0	1	1	1	0	0	0	0
*Elymus repens*	0	0	0	1	0	0	0	1	0	0	0	0	0	0	0
*Equisetum arvense*	0	1	1	1	0	0	0	1	0	0	1	0	1	1	0
*Erigeron annuus*	1	1	0	1	1	1	0	1	1	1	1	1	1	1	0
*Eruca sativa*	0	0	0	0	0	0	0	1	0	0	0	0	0	0	0
*Erysimum cheiranthoides*	1	0	0	1	0	0	0	0	0	0	0	0	0	0	0
*Euphorbia cyparissias*	0	0	0	1	1	0	0	1	0	0	0	1	0	0	0
*Euphorbia spec*.	0	0	0	0	1	0	0	0	0	0	0	0	0	0	0
*Festuca arundinaceae*	1	0	0	1	1	1	0	1	0	0	0	0	0	1	0
*Festuca ovina agg*.	0	0	0	0	0	0	0	1	1	1	1	1	0	0	0
*Festuca pratensis*	0	0	0	1	0	0	0	0	0	0	0	0	0	0	0
*Festuca rubra agg*.	0	0	0	1	1	1	1	0	0	0	0	0	0	0	0
*Filipendula ulmaria*	0	0	1	0	0	0	0	0	0	0	0	0	0	0	0
*Filipendula vulgaris*	0	0	0	0	1	1	0	0	0	1	1	0	0	0	0
*Galium mollugo agg*.	1	1	1	1	1	1	1	1	1	1	1	1	1	1	1
*Galium verum*	1	1	0	1	1	1	1	1	1	0	1	0	1	0	0
*Genista tinctoria*	0	0	0	1	0	0	1	0	0	0	0	0	0	0	0
*Geranium dissectum*	0	0	0	0	0	0	0	0	0	0	0	1	0	0	0
*Geranium palustre*	0	0	0	0	0	0	0	1	0	0	0	0	0	0	0
*Geranium pratense*	1	0	1	0	0	0	0	0	0	0	0	0	0	0	0
*Glechoma hederaceae*	0	0	0	0	0	0	0	1	0	1	0	0	0	1	0
*Helianthemum nummularium*	1	0	0	0	0	0	0	0	0	1	0	0	0	0	0
*Heracleum sphondylium*	1	1	1	1	1	0	0	1	0	1	0	0	1	0	0
*Hieracium pilosella*	1	0	0	1	0	1	1	1	0	1	1	0	0	0	0
*Hieracium spec*.	0	0	0	0	0	0	1	0	0	0	0	0	0	0	0
*Holcus lanatus*	1	1	1	1	1	1	1	1	0	1	0	1	1	1	1
*Hypericum perforatum*	1	1	1	1	0	0	1	1	0	1	1	1	0	0	0
*Hypochoeris radicata*	0	1	0	1	1	1	1	1	1	1	0	1	0	1	1
*Juncus effusus*	0	0	1	0	0	0	0	0	0	0	0	0	0	0	0
*Juncus tenuis*	0	0	0	0	0	0	0	0	0	0	0	0	0	0	1
*Knautia arvensis*	1	1	1	1	1	1	1	1	1	1	1	1	1	0	0
*Lathyrus pratensis*	1	1	1	1	1	1	0	1	0	1	0	1	1	1	1
*Leontodon hispidus*	1	0	0	0	1	1	1	1	1	1	1	1	0	1	0
*Leucanthemum vulgare*	1	1	0	1	1	1	1	1	1	1	1	1	1	1	1
*Linaria vulgaris*	1	0	0	0	0	0	0	0	0	0	0	0	0	0	0
*Linum cartharticum*	1	1	0	1	1	0	0	0	1	0	1	1	1	0	0
*Linum perenne*	0	0	0	0	0	0	0	0	0	1	0	0	0	0	0
*Lolium perenne*	0	0	1	0	0	0	1	0	0	0	0	0	0	0	1
*Lotus corniculatus*	1	1	1	1	1	1	1	1	1	1	1	1	1	1	1
*Luzula multiflora*	1	0	1	0	0	0	1	0	0	0	0	0	0	0	0
*Lychnis flos‐cuculi*	0	0	1	1	1	0	1	0	0	0	0	0	0	1	1
*Lychnis viscaria*	1	0	0	0	0	0	1	0	0	0	0	0	0	0	0
*Lysimachia nummularia*	0	0	0	0	0	0	0	1	0	1	0	0	0	0	0
*Lysimachia vulgaris*	1	0	0	0	0	0	0	1	0	1	0	0	0	0	0
*Malva moschata*	0	0	0	1	0	0	1	0	0	0	0	0	0	0	0
*Medicago falcata*	0	1	0	1	1	1	1	1	1	1	1	1	0	0	0
*Medicago lupulina*	1	0	0	1	1	1	1	1	1	1	1	1	0	0	0
*Medicago sativa*	0	0	0	0	0	0	0	0	0	1	0	0	0	0	0
*Melilotus officinalis*	0	0	0	0	0	0	0	1	0	0	0	0	0	0	0
*Mentha arvensis*	0	0	0	1	0	0	1	0	0	0	0	0	0	0	0
*Mentha longifolia*	0	0	0	0	0	0	0	0	0	1	0	0	0	0	0
*Molinea caerulea*	1	0	0	0	0	0	0	0	0	0	0	0	0	0	0
*Ononis spinosa*	0	0	0	1	1	1	0	1	0	1	1	1	0	0	0
*Origanum vulgare*	0	1	0	1	1	1	1	0	1	0	1	0	0	0	0
*Orobanche gracilis*	0	1	0	1	0	1	1	0	1	0	1	1	0	1	1
*Peucedanum oreoselinum*	0	0	0	0	1	1	0	0	0	0	0	0	1	0	0
*Phleum pratense*	0	1	1	0	0	1	1	0	0	0	0	0	1	1	0
*Picris hieracioides*	0	1	0	0	0	0	0	0	0	0	1	0	0	0	0
*Pimpinella major*	0	1	1	1	1	1	0	1	1	0	1	1	0	1	0
*Pimpinella saxifraga*	0	0	0	1	1	1	1	1	1	1	1	0	0	0	0
*Plagionium affine*	0	0	0	0	1	0	0	0	0	0	0	0	0	0	0
*Plantago lanceolata*	1	1	1	1	1	1	1	1	1	1	1	1	1	1	1
*Plantago media*	1	0	0	1	1	1	1	1	1	1	1	1	1	1	0
*Poa annua*	0	0	0	0	0	0	0	1	0	1	0	0	0	0	0
*Poa pratensis*	1	1	1	1	1	1	1	1	1	1	1	1	1	1	1
*Poa trivialis*	0	1	1	1	0	0	1	1	0	1	0	1	1	1	1
*Polygala vulgaris*	1	0	0	1	0	0	1	0	0	0	1	0	0	0	0
*Potentilla anserina*	0	0	1	0	0	0	0	0	0	0	0	0	0	0	0
*Potentilla argentea*	0	1	0	1	0	0	0	0	0	0	0	1	0	0	0
*Potentilla erecta*	1	0	0	0	0	0	0	0	0	0	0	0	0	0	0
*Potentilla recta*	0	1	0	0	0	0	0	0	0	0	0	1	0	0	0
*Potentilla reptans*	0	1	0	1	1	1	1	1	0	1	0	1	1	1	0
*Primula elatior*	1	0	0	1	0	1	0	0	0	1	0	0	1	0	0
*Prunella grandiflora*	0	0	0	0	0	?	1	0	0	0	0	0	0	0	0
*Prunella vulgaris*	1	1	1	1	1	1	1	1	1	1	1	1	0	1	1
*Ranunculus acris*	1	1	1	1	1	1	1	1	1	1	1	1	1	1	1
*Rhinantus minor*	1	1	1	1	1	1	1	1	1	1	1	1	1	1	1
*Rhythidiadelphus squarrosus*	0	0	0	1	0	0	1	0	0	0	0	0	0	0	0
*Rumex acetosa*	1	1	1	1	1	1	0	1	0	1	0	1	0	0	0
*Rumex crispus*	0	1	1	0	0	0	0	0	0	0	0	1	1	1	1
*Salvia pratensis*	1	1	0	1	1	1	1	1	1	1	1	0	0	0	0
*Sanguisorba minor*	0	0	0	0	1	0	0	0	0	0	0	0	0	1	0
*Sanguisorba officinalis*	1	0	1	0	1	1	0	1	1	1	1	1	1	0	0
*Scabiosa columbaria*	1	0	0	1	1	1	0	1	1	1	1	1	1	0	0
*Scleropodium purum*	0	1	0	1	0	0	1	0	0	1	0	1	0	0	0
*Sedum acre*	0	0	0	0	0	1	0	0	0	0	0	0	0	0	0
*Senecio jacobaea*	0	0	0	1	1	1	1	0	0	0	1	0	0	0	0
*Seseli libanotis*	0	0	0	0	1	1	0	0	0	0	0	0	0	0	0
*Silene vulgaris*	1	1	1	1	1	1	1	1	1	1	1	0	0	1	1
*Stellaria graminea*	1	0	1	0	0	0	0	0	0	1	0	1	1	0	0
*Symphytum officinale*	0	0	0	0	1	1	0	1	0	0	0	0	1	0	0
*Tanacetum vulgare*	0	1	0	1	1	0	1	0	1	1	0	0	0	0	0
*Taraxacum officinale*	1	0	1	1	0	1	1	1	1	1	1	0	1	1	1
*Thuidium tamariscinum*	0	0	0	0	0	0	1	0	0	1	0	0	0	0	0
*Thymus pulegioides*	1	0	0	0	0	0	1	0	1	1	0	0	0	0	0
*Tragopogon pratensis*	1	1	0	1	1	1	0	1	1	1	1	0	0	0	0
*Trifolium campestre*	0	1	0	1	0	1	1	0	0	0	0	0	0	0	0
*Trifolium dubium*	0	0	1	0	0	0	1	1	0	1	0	1	0	1	1
*Trifolium medium*	1	1	1	1	1	1	1	0	0	0	0	0	0	0	0
*Trifolium montanum*	0	0	0	0	1	1	0	1	0	1	0	0	0	0	0
*Trifolium pratense*	1	1	1	1	1	1	1	1	1	1	1	1	1	1	1
*Trifolium repens*	0	1	1	1	1	1	1	1	0	1	0	1	0	1	1
*Trisetum flavescens*	1	1	1	1	1	1	1	1	0	1	1	1	1	1	1
*Tussilago farfara*	0	0	0	0	0	0	0	1	0	0	0	0	0	0	0
*Verbascum lychnitis*	0	0	0	0	0	0	0	0	1	1	1	0	0	0	0
*Veronica chamaedris*	1	0	1	1	1	1	1	1	0	1	0	1	0	1	1
*Vicia cracca*	0	0	0	1	1	1	0	1	0	1	0	0	1	1	0
*Vicia hirsuta*	0	0	0	0	0	0	0	0	0	0	0	1	0	1	0
*Vicia sativa*	0	0	0	0	1	1	0	0	0	0	0	0	1	0	0
*Vicia sepium*	0	0	1	0	0	0	0	0	0	1	0	0	1	0	0
*Vicia tetrasperma*	0	0	0	0	0	0	0	0	0	0	0	0	0	1	0

**TABLE A2:** Molecular variance within and among populations of *K. arvensis* calculated in different analyses of molecular variance (AMOVA) based on 127 AFLP fragments

	*df*	SS	MS	%	Φ_PT_	*p*
Molecular variation among all populations						
Among Pops	11	168.25	15.30	4.01	0.040	.001
Within Pops	170	1,592.52	9.37	95.99		
Molecular variation among populations at source and restoration sites						
Among site type	1	18.26	18.26	0.46	0.043	.001
Among Pops	10	149.99	15.00	3.80		
Within Pops	170	1,592.52	9.37	95.74		
Molecular variation among populations at source sites						
Among Pops	2	34.760	17.380	5.55	0.055	.001
Within Pops	44	398.304	9.052	94.45		
Molecular variation among populations at restoration sites						
Among Pops	8	105.417	13.177	2.69	0.027	.001
Within Pops	126	1,173.768	9.316	97.31		
Molecular variation among single transfers:						
Transfer 1: S1 versus R1.1						
Among Pops	1	18.24	18.24	6.85	0.069	.003
Within Pops	29	247.30	8.53	93.15		
Transfer 2: S1 versus R1.1						
Among Pops	1	12.08	12.08	1.29	0.013	.137
Within Pops	29	291.40	10.05	98.71		
Transfer 2: S2 versus R2.2						
Among Pops	1	15.78	15.78	4.08	0.041	.003
Within Pops	30	281.56	9.39	95.92		
Transfer 2: S2 versus R2.3						
Among Pops	1	10.67	10.67	0.45	0.004	.352
Within Pops	29	289.27	9.97	99.55		
Transfer 3: S3 versus R3.1						
Among Pops	1	11.73	11.73	1.98	0.020	.076
Within Pops	28	252.13	9.00	98.02		
Transfer 3: S3 versus R3.2						
Among Pops	1	13.25	13.25	2.81	0.028	.023
Within Pops	29	265.46	9.15	97.19		
Transfer 3: S3 versus R3.3						
Among Pops	1	18.70	18.70	6.15	0.061	.001
Within Pops	28	264.13	9.43	93.85		
Transfer 3: S3 versus R3.4						
Among Pops	1	20.58	20.58	7.39	0.074	.001
Within Pops	27	257.83	9.55	92.61		
Transfer 3: S3 versus R3.5						
Among Pops	1	14.34	14.34	3.78	0.038	.004
Within Pops	27	246.76	9.14	96.22		

Note

Levels of significance (*p*‐values) are based on 999 iteration steps.

Abbreviations: %, proportion of genetic variance; *df*, degree of freedom; MS, mean squares; SS, sum of squares.

**TABLE A3:** Molecular variance within and among populations of *P. lanceolata* calculated in different analyses of molecular variance (AMOVA) based on 122 AFLP fragments

	*df*	SS	MS	%	Φ_PT_	*p*‐value
Molecular variation among all populations						
Among Pops	14	191.76	13.70	2.21	0.022	.001
Within Pops	213	2,172.93	10.20	97.79		
Molecular variation between populations at source and restoration sites						
Among Site Class	1	11.78	11.78	0.00	0.021	.001
Among Pops	13	180.37	13.87	2.32		
Within Pops	213	2,172.54	10.20	97.68		
Molecular variation among populations at source sites						
Among Pops	3	40.95	13.65	2.05	0.021	.008
Within Pops	59	605.40	10.26	97.95		
Molecular variation among populations at restoration sites						
Among Pops	10	139.04	13.90	2.38	0.024	.001
Within Pops	154	1,567.53	10.18	97.62		
Molecular variation among single transfers						
Transfer 1: S1 versus R1.1						
Among Pops	1	10.98	10.98	0.12	0.002	.385
Within Pops	29	309.02	10.66	99.80		
Transfer 1: S1 versus R1.2						
Among Pops	1	8.62	8.62	0.00	0.008	.690
Within Pops	29	284.22	9.80	100.00		
Transfer 2: S2 versus R2.1						
Among Pops	1	15.41	15.41	3.04	0.030	.014
Within Pops	30	307.94	10.26	96.96		
Transfer 2: S2 versus R2.2						
Among Pops	1	13.59	13.59	2.22	0.022	.048
Within Pops	30	299.31	9.98	97.78		
Transfer 2: S2 versus R2.3						
Among Pops	1	17.88	17.88	4.81	0.048	.001
Within Pops	30	296.38	9.88	95.19		
Transfer 3: S3 versus R3.1						
Among Pops	1	18.45	18.45	4.54	0.045	.001
Within Pops	26	288.55	11.10	95.46		
Transfer 3: S3 versus R3.2						
Among Pops	1	16.63	16.63	3.42	0.034	.012
Within Pops	28	304.27	10.87	96.58		
Transfer 3: S3 versus R3.3						
Among Pops	1	16.49	16.49	3.28	0.033	.018
Within Pops	27	298.61	11.06	96.72		
Transfer 3: S3 versus R3.4						
Among Pops	1	14.36	14.36	2.19	0.022	.061
Within Pops	26	284.71	10.95	97.81		
Transfer 3: S3 versus R3.5						
Among Pops	1	16.51	16.51	4.02	0.040	.004
Within Pops	29	290.46	10.02	95.98		
Transfer 4: S4 versus R4.1						
Among Pops	1	12.69	12.69	1.97	0.020	.057
Within Pops	30	288.00	9.60	98.03		

Note

Levels of significance (*p*‐values) are based on 999 iteration steps.

Abbreviations: %, proportion of genetic variance; *df*, degree of freedom; MS, mean squares; SS, sum of squares.
